# Supplementary figures and images for: Fibrotic expression profile analysis reveals repurposed drugs with potential anti-fibrotic mode of action
Source: PLoS One. 2021 Apr 7;16(4):e0249687. doi: 10.1371/journal.pone.0249687 (PMC8026018; doi:10.1371/journal.pone.0249687)

Height

0 1 2 3 4 5 6 7

Soergel Distance

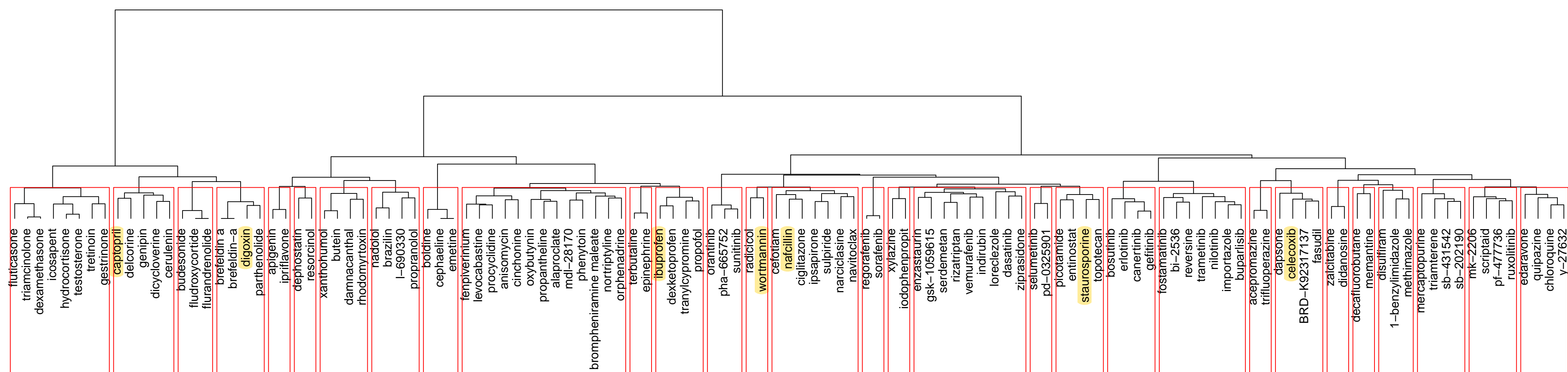

as.dist(distance)  
hclust (\*, "ward.D")

Supplement: S1 Fig — (PDF) [file pone.0249687.s001.pdf]
